# Factors predicting the outcome of intravenous thrombolysis in stroke patients before rt-PA administration

**DOI:** 10.22088/cjim.10.4.424

**Published:** 2019

**Authors:** Masoud Mehrpour, Motahare Afrakhte, Seyedeh Fahimeh Shojaei, Ahmad Sohrabi, Rezan Ashayeri, Sara Esmaeili, Maryam Bahadori

**Affiliations:** 1 Department of Neurology, Firoozgar Hospital, Iran University of Medical Sciences, (IUMS), Tehran, Iran; 2 Firoozgar Clinical Research and Development Center (FCRDC), Iran University of Medical Sciences (IUMS) , Tehran, Iran; 3 Infectious disease research center, Golestan University of Medical Sciences, Gorgan, Iran

**Keywords:** Ischemic stroke, Thrombolytic therapy, rt-PA

## Abstract

**Background:**

To determine whether it is possible to predict intravenous thrombolytic therapy (IVT) outcome after 3 months in acute ischemic stroke patients who are candidate to receive recombinant tissue plasminogen activator (rt-PA), before rt-PA administration based on their risk factors and some available laboratory results.

**Methods:**

We enrolled 118 ischemic stroke patients who were treated with standard dose of Alteplase in our hospital. Baseline characteristics, door-to-needle time (DTN), onset-to-treatment time (OTT), the National Institute Health Stroke Scale (NIHSS), systolic and diastolic blood pressure on admission, history of diabetes, hypertension, dyslipidemia, coronary artery disease (CAD), previous ischemic stroke, atrial fibrillation (AF), laboratory results were retrospectively collected. The modified Rankin Scale (mRS) was recorded after 3 months of admission and patients were divided into good (mRS 2) and poor (mRS>2) outcome groups. Chi-square test and t-test were used for categorical and continuous variables, respectively. Predictors for outcome after 3 months were studied by multivariable logistic regression.

**Results:**

Good outcome was seen in 60 (51%) patients and poor outcome was seen in 58 (49%) patients. Significant predictors for outcome at 3 months according to multivariable regression analysis were NIHSS score (odds ratio [OR], 0.61; 95% confidence interval [CI], 0.498-0.750; p<0.001), SBP (OR, 0.95; 95% CI, 0.925-0.991; P=0.01), AF (OR, 0.09; 95% CI, 0.013- 0.708; P=0.02), CAD (OR, 17.08; 95% CI, 0.013-0.708; p=0.003).

**Conclusion:**

Higher NIHSS score, higher SBP on admission, AF and history of CAD could be the independent predictors of outcome after IVT in acute ischemic stroke patients.

Intravenous recombinant tissue plasminogen activator (IV rt-PA) in proper patients within 4.5 hours of symptom onset, is the main treatment in the acute phase of ischemic stroke, which can improve functional outcome significantly (1). However, not all patients experience a good functional outcome with intravenous thrombolytic therapy (IVT). Each ischemic stroke patient can be different from the other one considering some factors like baseline conditions and presence of stroke risk factors which may influence the outcome of IVT. Ability to predict good outcome shortly after admission can play an important role in decision making for the best treatment plan. It is also important for patients and their caregivers to have more realistic expectations of IVT, and maybe it can alter their decision to choose the treatment. There are discrepancies in the literature regarding prediction of patients’ outcomes who are going to receive rt-PA therapy.

For instance, it has been reported in some researches that HTN, DM, hyperlipidemia, AF, CHD are not prognostic factors for functional outcome (2-4), while in some other studies dyslipidemia, high BS (5), AF, CHD (6) are related to patients’ outcome. In addition, in some reports, ethnicity is found to be an important predictor of stroke outcome (7, 8). The aim of the present study is to investigate the predictors of IVT good outcome in Iranian patients with ischemic stroke before rt-PA administration. 

## Methods

**Study participants: **This is a retrospective study which was done at Firoozgar University Hospital, one of the major stroke referral centers in Tehran, Iran. A total of 118 patients treated with IVT (Alteplase) between June 2015 and November 2017 were enrolled in this study. Initially, there were 138 patients who had received IVT in our center during the mentioned time period, however 118 of them met the criteria to be evaluated in this study. Upon arrival in the hospital, medical and medication history was taken to evaluate the risk of bleeding in each patient. A quick neurological examination was performed by neurology team and stroke severity was assessed by using the National Institutes of Health Stroke Scale (NIHSS) score. Non-contrast CT scan and following laboratory tests were requested immediately: Complete blood count test, Blood sugar, Creatinine and coagulation tests (INR, PT, PTT). Exclusion and inclusion criteria for IVT was defined based on AHA/ASA guideline. Eligible patients admitted within 4.5 hours of symptom onset, received 0.9 mg/kg of rt-PA to the maximum dose of 90 mg intravenously (10% bolus, 90% infusion in 1 hour). All patients were admitted to stroke unit care after receiving IV-rt-PA for further management and rehabilitation. The exclusion criteria for this study were as following: 1- Patients who did not receive all calculated rt-PA dose completely due to uncontrolled blood pressure after rt-PA administrationor any other reasons, 2- Those who received rt-PA beyond 4.5 hours of symptom onset, 3- Patients whose final diagnosis was not acute ischemic stroke despite receiving rt-PA (stroke mimics), 4- Patients who were not available for follow up or were not content for enrolling in this study.

**Data collecting: **Hospital documents were retrospectively reviewed by a physician who was blind to patients' outcomes to collect the following data: demographic characteristics, initial systolic and diastolic blood pressure, symptom onset to needle time (OTN), door to needle time (DTN), stroke severity based on national institutes health stroke (NIHSS) score, past medical history of diabetes mellitus, hypertension, dyslipidemia, coronary artery disease, and past history of ischemic stroke. Those patients who had arterial fibrillation (AF) on cardiac monitoring at the time of admission or were known cases of AF were also determined as AF patients. The study was approved by the Iran University of Medical Sciences Ethics Committee.

**Outcome measurement: **Three months after the admission of modified Rankin Scale (mRS) as the outcome measurement was evaluated by the telephone interview. Each interview lasted 10 to 20 minutes with patients or their primary caregivers to determine the score. Patients were classified into 2 groups: good outcome (mRS 0 to 2) and poor outcome (mRS 3 to 6). 

**Statistical analysis: **Continuous variables are expressed as meanSD, and categorical variables are expressed as counts (n) and percentage (%). Univariate analysis was done using t-test and chi-square test for continuous and categorical variables, respectively. Multivariable logistic regression was applied to determine the independent association between good outcome (mRS 0-2) or poor outcome (mRS 3-6) and clinical factors and laboratory results. The significance level was determined at p-value < 0.05. All analyses were done by SPSS Version 16.

## Results

During the study period, between June 2015 and November 2017,133 patients received IVT in our hospital. Of those, 118 were included in this study. There were 78 (66%) males and 40 (34%) females in this study, and the age range was 31-90 years with median 67.5. After a time period of 3 months, good outcome (mRS 0-2) was seen in 60 (50.8%) patients and poor outcome in 58 (49.2%) patients. Table 1 compares the clinical characteristics and laboratory results of good outcome group with poor outcome group. 43 (55%) males had good outcome and 35 (45%) had poor outcome. 17 (42%) females had good outcome and 23 (57%) had poor outcome. Age (P=0.002), NIHSS (P<0.001), AF (P=0.001) were significant in univariate analysis. After entering all variables in multivariable logistic regression, following factors were significantly related to mRS at 3 months: NIHSS score (odds ratio [OR],0.61;95% confidence interval [CI], 0.498-0.750; P<0.001), SBP (OR, 0.95; 95% CI, 0.925-0.991; P=0.01), AF (OR, 0.09; 95% CI, 0.013- 0.708; P=0.02), CAD (OR,17.08; 95% CI, 0.013-0.708; P=0.003) (table 2).

**Table-1:** Clinical features and laboratory findings in good outcome group vs poor outcome group

**Variable**	**Total** **N = 118**	**Good outcome** **N = 60**	**Poor outcome** **N = 58**	**p-value**
Age, mean (±SD)	66.12(13.46)	62.40(12.84)	69.96(13.11)	0.002
SBP (mmHg)	154.35(26.55)	149.80(26.17)	159.06(26.34)	0.058
DBP (mmHg)	93.66(21.10)	90.68(13.56)	96.75(26.54)	0.123
DTN (min)	53.21(35.28)	49.58(33.05)	56.96(37.35)	0.258
STN (min)	144.90(62.63)	143.26(63.79)	146.60(61.92)	0.774
NIHSS	11.10(5.06)	7.96(4.23)	14.34(3.61)	<0.001
PLT	219.56(82.18)	216.01(70.22)	223.18(93.28)	0.639
Initial plasma glucose (mg/dL)	169.95(78.15)	171.50(87.32)	168.36(68.11)	0.828
Creatinine	1.15(0.43)	1.09(0.23)	1.22(0.57)	0.117
Sex: male (%)	78(66.10)	43(55.12)	35(44.87)	0.194
Previous stroke	25(21.20)	12(48)	13(52)	0.748
Hypertension	82(69.50)	42(51.21)	40(48.78)	0.903
Coronary artery disease	55(46.60)	30(54.54)	25(45.45)	0.453
Diabetes mellitus	41(34.70)	22(53.65)	19(46.34)	0.656
Dyslipidemia	28(23.70)	15(53.57)	13(46.42)	0.741
Atrial fibrillation	24(20.30)	5(20.83)	19(62.67)	0.001
Smoking	25(21.20)	14(56)	11(44)	0.562

Note: SBP: Systolic blood pressure, DBS: Diastolic blood pressure, DTN: door to needle, STN: symptom to needle, NIHSS: National Institute Health Stroke Scale.

**Table-2:** Logistic regression analysis predicting outcome of intravenous thrombolytic therapy for acute ischemic stroke patients

	**Adjusted estimation**			**Crude estimation**		
**Variable**	**OR**	**(95% CI)**	**P**	**OR**	**(95% CI)**	**P**
Sex	0.415	(0.078 - 2.210)	0.303	.602	(.279 - 1.299)	.196
Previous stroke	0.904	(0.179 - 4.564)	0.902	.865	(.358 - 2.094)	.748
Hypertension	1.473	(0.282 - 7.693)	0.646	1.050	(.480 -2.299)	.903
Coronary artery disease	17.085	(2.544 - 114.722)	0.003	1.320	(.639 -2.726)	.453
Diabetes mellitus	0.578	(0.102 - 3.283)	0.536	1.188	(.556 -2.539)	.656
Dyslipidemia	0.403	(0.073 - 2.214)	0.296	1.154	(.493 -2.699)	.741
Smoking	0.345	(0.050 - 2.361)	0.278	1.300	(.535 -3.161)	.562
Atrial fibrillation	0.095	(0.013 - 0.708)	0.022	.187	(.064 - .542)	.002
Age	0.963	(0.916 - 1.011)	0.132	1.046	(1.015 -1.078)	.003
SBP	0.957	(0.925 - 0.991)	0.012	1.012	(.997 -1.027)	.110
DBP	1.000	(0.968 - 1.033)	0.999	1.014	(.995 -1.034)	.142
DTN	0.992	(0.970 - 1.013)	0.441	1.005	(.995 -1.016)	.319
OTN	1.000	(0.988 - 1.013)	0.962	1.001	(.995 -1.007)	.762
NIHSS	0.611	(0.498 - 0.750)	< 0.001	1.431	(1.266 -1.618)	.000
PLT	0.993	(0.986-1.001)	0.082	1.001	(.997-1.006)	.637
Initial plasma glucose	1.002	(0.993-1.011)	0.624	.999	(.995-1.004)	.827
Creatinine	0.128	(0.010-1.692)	0.118	2.427	(.757-7.784)	.136

## Discussion

The aim of this study was to evaluate the outcome predictors among Iranian patients shortly after admission. We found that 51% of our patients experienced good outcome (mRS2), and 49% experienced poor outcome (mRS 3-6). In current study, it was found that NIHSS score, systolic blood pressure, AF and CAD were significantly related to functional outcome among our patients who underwent IVT. 

We chose mRS 2 as the good outcome score because at this score patients are independent and can do their routine daily activities without any help. However, in some studies, other cutoffs were defined as good outcome (9). Previously the stroke-thrombolytic predictive instrument (TPI), multimodal outcome score for stroke thrombolysis (MOST) and DRAGON score have been introduced to predict outcome in patients who underwent IVT. Each of these scores has different criteria to predict the outcome (9-11). 

Our data suggest that higher NIHSS score and high systolic blood pressure at admission, history of AF and CAD were associated with poor outcome (mRS 3-6). Although intracranial hemorrhage (ICH) especially symptomatic ICH as a potential adverse effect of rt-PA can influence the outcome of IVT, we did not consider ICH in our study because the aim of this study was to determine predictive factors before the administration of rt-PA.

**Effect of NIHSS on outcome: **NIHSS is an examination scale including 15 items for assessment of stroke severity. The total score is 0 to 42, the higher the score, the more severe is the stroke. Other studies came to the conclusion that NIHSS had good reliability and validity to determine the severity of neurologic deficit in stroke patients (12, 13). 

MOST and DRAGON scores, and also TPI score (only for poor outcome prediction), use NIHSS score to predict functional outcome. Our findings were consistent with other studies in which higher admission NIHSS score was associated with more unfavorable outcome, in both groups of patients who received IVT or not (14-18). In a recent study, NIHSS 15 was determined as a long term good prognostic factor in stroke patients who had received rt-PA at least 1 year before assessment. We should also consider this fact that NIHSS may not be a good tool to assess the severity of stroke in posterior circulation strokes (19, 20). It implies that if in our study the exact territory of stroke had been defined, probably the NIHSS score was less predictive for functional outcome.

**Effects of Systolic Blood Pressure on outcome: **The correlation of baseline systolic blood pressure (SBP) with outcome of ischemic stroke has been discussed in several studies. Some studies suggested that higher SBP is associated with poor outcome outcome (21, 22) or good outcome (23). 

Some other studies found a U-shape relationship between SBP and outcome of ischemic stroke. High systolic blood pressure can cause more brain edema, stroke recurrence or intracranial pressure, while low blood pressure can extend the infarct area by lowering blood perfusion, increasing the risk of cardiovascular disease or cerebral reinfarction (24, 25).

In one study with 17398 participants from International Stroke Trial, there was a U-shape association between baseline SBP and death or dependency after 6 months. This study showed that those patients whose baseline SBP was between 140-179 had the better outcome than other patients, and the best outcome was in those whose SBP was around 150 mmHg. In this study, almost 5% of patients had SBP<120 which was associated with the poor outcome due to cardiovascular events (25).

 Of interest, in our study, there were also 6 (5.1%) patients with baseline SBP lower than 120 mmHg, among them 2 patients had poor outcome, which both of them had the history of ischemic heart disease and expired after about 2 months due to myocardial infarction. In a more recent study in China in which only those stroke patients were assessed who underwent thrombolytic therapy within 4.5 hours of their symptoms, it was noted that lower baseline SBP had a significant independent relationship with favorable outcome which was defined as mRS 0-1 (6). 

**Effect of Atrial Fibrillation and Coronary Artery Disease on outcome: **AF is a well-known cardiac risk factor for ischemic stroke. Ischemic stroke can be the initial presentation in AF patients or it can occur in anticoagulated AF cases. In our study population, 24 (20%) patients were defined as AF patients, either chronic or new cases of AF. It has been shown in some studies that AF patients have a greater risk of having poor outcome after stroke (25-27).

Probably AF increases the risk of the second stroke (28). In addition, the malignant massive stroke which has the higher risk of mortality is more frequent in cardio-embolic origin of stroke (6). The worse outcome of ischemic stroke among AF patients has been also referred to the higher risk of hemorrhage in some studies (29). Cetiner et al. reported that in their study, AF patients had even better functional outcome, maybe because this fact that cardiac originating embolisms are richer in fibrin compare to those which are originating from atherosclerotic plaques which are full of pellets, so embolic thromboses are more soluble while contacting with rt-PA (30). 

A recent study has suggested that poor outcome is higher in the elderly group of AF patients, and younger patients may experience better outcome (31). 

By coronary artery disease, we mean the history of the acute coronary syndrome and angina pectoralis. It has been shown in some studies that patients with the history of MI have more poor outcome after ischemic stroke (32). In our study, the presence of CAD was significantly different between good outcome patients and poor outcome ones. Stroke and coronary heart disease have common risk factors and pathophysiology. 

For example, AF as a predictor of poor outcome in stroke patients is also more prevalent in those who suffer from CAD. This is a limitation of real clinical scenario studies including ours. CAD and/or AF are independent predictors in some scores for overall ischemic stroke patients' outcome (32-34). 

But it is not included in any of the mentioned scores which assess outcome in patients who underwent IVT. Further studies will be needed to determine whether the presence of CAD or AF can be predictive of stroke outcome of patients who underwent IVT or not. Although in many studies advancing age has an important impact on thrombolytic therapy outcome (8-10). We did not find any association between age and outcome of IVT among our study patients.

**Study limitations: **The limitation in this study is the retrospective nature of the study. We did not consider the radiologic features of each patient including the location and size of ischemia in the current study. We did not include previous medication and lipid profile in our study due to a considerable number of missing data. Further prospective studies with more patients are warranted to confirm our findings.

In conclusions, according to our study in the emergency setting and before administration of rt-PA, higher NIHSS, higher initial systolic blood pressure and the presence of CAD history or AF are important factors which can predict functional outcome and dependency status 3 months after ischemic stroke.
